# Mapping Colombians’ positions on sentencing for substance offenses

**DOI:** 10.1186/s13011-022-00485-z

**Published:** 2022-07-28

**Authors:** Daniel del Rio Forero, Claudia Pineda Marín, María Teresa Muñoz Sastre, Lonzozou Kpanake, Etienne Mullet

**Affiliations:** 1 CERPPS, Maison de la recherche, Federal University of Toulouse, 5 allées Antonio Machado, 31058 Toulouse cedex 9, France; 2 Konrad Lorenz University, Cra. 9 Bis #No. 62 - 43, Bogotá, Colombia; 3 University of Québec – TELUQ, 5800, rue Saint-Denis, Bureau 1105, Montréal, Québec H2S 3L5 Canada; 4 Institute of Advanced Studies (EPHE), 17 bis, rue Quefes, 31830 Plaisance du Touch, France

**Keywords:** Drug offences, Personal positions, Sentencing, Colombia

## Abstract

**Background:**

In Colombia, a person caught in possession of an illicit substance is not judicially sanctioned as long as the quantity does not exceed the maximum allowable amounts. Given that the public is divided on the appropriateness of this policy, an examination of the various public positions was undertaken.

**Method:**

A convenience sample of 302 adults were presented with 48 vignettes depicting a situation of everyday life easily recognizable by all in Colombia; that of a male person who is apprehended in the street by the police because he is suspected of illicit trafficking. These vignettes were created by orthogonal variation of four factors: Type of substance, amount of substance, type of charge against the offender, and offender’s age.

**Results:**

Through cluster analysis, six qualitatively different positions were found. These positions corresponded to three distinct, classical philosophies (a) a libertarian, free-market philosophy – punishment should never be extremely severe because the trade in psychotropic substances is a trade like any other (6%), (b) a moralistic, conservative philosophy – punishment should always be extremely severe except perhaps in certain cases (52%), and (c) a progressive, human rights-inspired philosophy – punishment should always be proportional to the seriousness of the facts (42%).

**Conclusion:**

Half of Colombian people supported a control policy according to which the use of psychotropic substances is considered profoundly immoral. Most of the other segment of the population express views similar to those of international organizations. It is therefore desirable that legislators rely on progressive international legislation to support domestic policies that are not strictly moralistic and conservative.

## Background

The objective of this study was to characterize precisely the diverse positions of Colombian citizens regarding the type of punishment that should be imposed on a person who has been arrested by the police while in possession of illicit substances. In Colombia, in 2019, it is estimated that about 8% of people aged 12 to 65 have used cannabis at least once, about 2% have used cocaine, and 0.1% have used heroin. The percentages of regular users of these substances (monthly use) are approximately 2, 0.3 and 0.01%, respectively [1]. For cannabis, the average age of initiation is about 18 years. About 55% of people feel that if they wanted it, it would be easy to get it and only 7% of people have never been openly offered it in their lives. In 2014, about 85,000 people were arrested for the crime of trafficking, manufacture or carrying of narcotics, 16,000 were convicted, and 200 were extradited [2].

In Colombia, as in other countries, governments have been concerned about the presence of illicit substances in their territory. This concern ranges from the planting of so-called illicit crops to drug trafficking. Multiple strategies have been put in place to control drug crops (e.g., aerial spraying of glyphosate, manual eradication, prosecution of growers) as well as to control drug trafficking. However, perspectives and legislation have oscillated between two poles: prohibitionist/repressive and progressive, i.e., focusing on social and cultural measures. In 1994, the Colombian Constitutional Court had already ruled (Ruling C-221) that the control of substances should be carried out with respect to the rights of people to autonomous development and, therefore, the consumption of personal doses should be decriminalized [3]. On the contrary, in 1999, under the Pastrana government, a strategy called Plan Colombia was implemented with the objective of totally prohibiting the use of drugs, even in small quantities [4]. In 2002, under the repressive-minded Uribe government, the Constitutional Court (Ruling C-689) nevertheless insisted on the importance of differentiating between trafficking, possession and consumption in court decisions [5]. In 2009, the Constitutional Court went a step further and ruled that the criminalization of drug use should be done in the spirit of public health and not in the spirit of criminalization [5]. Finally, from 2011 onwards, the policy of repression in the control of drug use gave way to a true public health perspective, which resulted in the decriminalization of the possession and consumption of personal doses and the concern for health care for users of illicit substances, instead of incarceration [3].

Currently, in Colombia, there are therefore no judicial sanctions against use of illicit substances, but the law has provided mechanisms that aim to control the internal trafficking of drugs in the country and to reduce the harm associated with drug use. Specifically, a person caught in possession of an illicit substance such as marijuana, hashish, cocaine (or its derivatives) or methaqualone is not judicially sanctioned as long as the quantity does not exceed the maximum allowable amounts, which are 20 g of marijuana, 5 g of hashish, 1 g of cocaine and 2 g of methaqualone. Otherwise, individuals can face prison sentences of 4 years to 30 years as well as economic sanctions [6–8].

In reality, it is difficult for police and judges to discern whether people who are caught with illicit substances are carrying them only for consumption or are engaged in micro-trafficking (distribution of drugs in small quantities to evade police prosecution). In 2017, 31% of the convictions with deprivation of liberty were for people who had only the maximum allowable amount on their person and for whom no evidence of trafficking could be established [9].

### Citizens’ views regarding sentencing for substance offenses

It may seem strange to examine citizens’ positions on a subject as complex as criminalization legislation related to substance offenses [10]. This legislation must take into account a multiplicity of extremely diverse factors such as considerations of public order (e.g., repression of violent criminality), considerations of public health (e.g., treatment of the sick), budgetary costs (e.g., prison management), or international relations (e.g., the need for harmonization of legislation at the regional or global level).

As previous studies in the United Kingdom have shown [11, 12], ordinary citizens, in their assessment of the level of punishment required for trafficking or substance use, (a) are likely to be overwhelmed by their emotions and call for disproportionate sentences, or (b) may be guided by their interests and, if they themselves feel implicated (e.g., regular users), propose sentences that are too lenient. It is likely that this finding also applies to many other publics, including the Colombian public.

However, a sentencing system that is completely at variance with citizens’ opinions may not be easily enforceable [13]. It is important to know the extent to which the current sentencing system differs from the views of citizens. Moreover, citizens’ positions on this issue are very likely to be diverse. They are not usually reduced to a point along a scale from unfavorable to favorable. They have a structure. Knowing these structures implies conducting a detailed characterization of them.

Kirby and Jacobson [14] conducted a study in England and Wales that examined citizens’ views (a) on the relative severity of different drug offenses, particularly possession, supply, and importation, (b) on the relevance of the type of drugs to the severity of drug offenses; and (c) on the aims of sentencing itself. They used a vignette technique and the six offenses used to create each story were possession of cannabis, small-scale supply of cannabis, large-scale supply of heroin, medium-scale importation of cocaine, medium-scale supply of crack, and large-scale importation of heroin. In other words, they manipulated three factors – the type of substance (cannabis, cocaine and heroin), the amount of substance (small or large), and the nature of the charge against the offender (possession, supply and importation) – and assessed their participants’ reactions to this manipulation.

Participants were sensitive to all three factors. Firstly, participants made a clear differentiation between possession charges and other types of drug charges. Their main argument was that possession and personal use only cause harm to oneself, while supply and importation result in harm to others. Secondly, cannabis-related offenses were largely considered to deserve less rigorous sentences than those related to any of the other drugs. Thirdly, the majority penalty for the supplier of mild quantities of crack and cocaine was 2 to 15 years in prison, while for the large-scale supplier of heroin it was 10 to 20 years. Kirby and Jacobson [14] also reported that the “phrase that was perhaps repeated more than any other in the focus group discussion was ‘he knows what he’s doing’.” This sentence suggests that the severity of sentences may also be related to the degree of personal maturity of transgressors.

Gritsenko and collaborators [15] explored Russian university students’ attitudes toward drug trafficking. They found that (a) 56% advocated “strict and very severe punishment (including death)”, regardless of the circumstances, (b) 27% felt that punishment should “take into account mitigating factors such as unemployment, the need to support a family”, and (c) 17% felt that punishment should “take into account objective measures of the crime committed (volume of sales, duration of trade, and others).”

Jorgensen [16] examined the opinions of US police officers regarding appropriate sentences for various drug offenses. More than 80% considered a minimum of 1 year in prison to be an appropriate sentence for people arrested for selling cocaine or heroin for profit. In the case of persons arrested for selling cannabis or using cocaine or heroin, only about 30% considered such a sentence to be appropriate. In the case of a person arrested for cannabis use, only 5% thought that such a sentence was appropriate. The very religious officers were harsher in their opinions than the non-religious ones.

### The present study

As noted above, this study sought to characterize in detail the diverse positions of Colombian citizens -- a population for which little empirical data on the subject is available -- regarding the type of punishment to be meted out to a person who has been arrested by the police while in possession of illegal substances. As also suggested above, these positions are likely to be extremely varied and each is likely to be complex and to have structure. They cannot be reduced to simple placements along a scale of sentencing severity. It is therefore necessary, if we are to understand these positions, to adopt a methodological approach that is sufficiently flexible to be able to capture this diversity and complexity.

This is why the present study was conducted according to an approach already used in other fields, whenever a fine characterization of public positions is desired [17–20]. A number of vignettes were created by orthogonal variation of the three factors considered by Kirby and Jacobson [14]: type of substance (cannabis, cocaine, or heroin), amount of substance, and type of charge against the offender (simple possession, sale to adults, sale to teens, canvassing at the school door). A fourth factor, also suggested by these authors, was considered: the age of the offender, minor or major. A situation of everyday life easily recognizable by all in Colombia was chosen; that of a male person who is apprehended in the street by the police because he is suspected of illicit trafficking.

Colombians’ positions on the severity of punishment in the various situations described in the vignettes are likely to be extremely varied. In a recent survey conducted in Bogotá on adults’ perspectives on possible drug control policies, no less than seven qualitatively different positions were identified [21]. The most common position (50% of participants) was that no control policy was adequate. These participants tend to believe that neither legalization nor prohibition of substances can address the psychological and social underlying causes of their use. Many Colombians tend to believe that the origins of the drug problem lie abroad, in wealthy economies where a significant portion of the population is willing to spend large amounts of money in exchange for small amounts of powder. For about half of the participants expressing this radical position, the only thing the government can do is to inform the public of the dangers of drug use. The second most common position (19%) was that a policy of complete prohibition was the only one that would be adequate (although half of the members of this group were willing to allow cannabis to be sold freely). The third one (14%) was similar except that participants were also considering as acceptable a policy of complete regulation by the government. For 8%, the only valid option was that the drug market should be free.

We therefore expected several qualitatively different positions to be expressed by the participants. The first expected position is that of the participants for whom as soon as a person is convicted of drug offence, this person should be sentenced in the most severe way possible, regardless of the circumstances. This position is based on the philosophy of the war on drugs. Gritsenko and collaborators [15] showed that this position is very common in Russia, even among students. A symmetrical position, probably in the minority, should also be found. Since for a certain percentage of Colombians the drug market should be free, no conviction should be incurred by anyone (except perhaps those canvassing at school gates).

Intermediate positions must also be found. For a significant number of people, the severity of the penalty should be proportionate to the danger they pose to others. Canvassing at school gates should be punished much more severely than being in possession of a single dose, especially if the substance in question is heroin and the seller is a mature adult rather than an underage youth. Kirby and Jacobson [14] showed that their participants clearly distinguished between possession charges and sales charges, between cannabis-related offenses and cocaine-related offenses, between supplying light amounts and supplying large amounts, and that they were sensitive to the degree of personal maturity of the offender.

Finally, for a certain percentage of participants, Colombia’s current law should apply in all cases. The Colombian Constitutional Court has made it clear that possession of a personal dose of any drug is decriminalized [8]. Therefore, possession of a small amount of cannabis (or cocaine, or heroin) should not result in a sanction, but the sale of illicit substances should be severely punished, regardless of the circumstances.

We also expected that the frequency of expression of these different positions would differ according to participants’ age, whether they had children, and their degree of religiosity. For example, if a position of the harshest possible punishment in all cases is evidenced, this position should be more frequently expressed among those who are older and have children [22, 23], or report a very high degree of religiosity [24].

## Method

### Participants

The participants in this study were a convenience sample of 302 adults (36% men) aged 18 to 85 years (*M* = 37.32, *SD* = 13.60) residing in Bogotá, Colombia. Their demographic characteristics are shown in Table 1. Some of the participants (*N* = 190) were approached in different districts of the city. They were requested to participate in the survey while they were walking on the main pedestrian sidewalks in their barrio, usually in nearby areas of public facilities, commercial centers, and the churches. The participation rate was 51%. The main explanation expressed for not taking part in the study was time constraints. The remaining participants (*N* = 112) were surveyed through internet, because of COVID-19 and the Colombian government’s mobility restrictions. A judge from the Civil Court of Bogota also agreed to participate.

**Table 1:** Demographic characteristics of the sample. Composition of the clusters

	Cluster						
Factor	Never Severe	Depends on Charge	No Sale	Except if Small	Always if Adult	Always Severe	Total
Age							
18-28 Years	9 (11)^ab^	41 (51)^a^	5 (6)	9 (11)^a^	13 (16)^a^	4 (5)^a^	81
29-35 Years	7 (9)	28 (37)^b^	2 (3)	17 (23)^a^	14 (19)	7 (9)^b^	75
36-49 Years	2 (2)^a^	30 (38)^c^	2 (2)^a^	14 (18)	23 (29)^a^	9 (11)^c^	80
50+ Years	1 (1)^b^	13 (20)^abc^	7 (11)^a^	10 (15)	16 (24)	19 (29)^abc^	66
Gender							
Male	9 (8)	41 (37)	7 (7)	15 (14)	30 (27)	8 (7)^a^	110
Female	10 (5)	71 (37)	9 (5)	35 (18)	36 (19)	31 (16)^a^	192
Socio-Economic Level							
Very Low	5 (10)	12 (24)^a^	2 (4)	7 (14)	13 (26)	11 (22)^a^	50
Low	3 (3)	43 (45)^a^	4 (4)	20 (21)	14 (15)^a^	12 (12)	96
High	6 (6)	39 (39)	8 (8)	11 (11)	27 (27)^a^	9 (9)^a^	100
Very High	5 (9)	18 (32)	2 (4)	12 (21)	12 (21)	7 (13)	56
Children							
No	16 (8)	85 (43)^a^	10 (5)	32 (16)	38 (19)	17 (9)^a^	198
Yes	3 (3)	27 (26)^a^	6 (6)	18 (17)	28 (27)	22 (21)^a^	104
Religious Involvement							
Very Low	7 (13)^a^	30 (58)^abc^	1 (2)	6 (12)	7 (13)	1 (2)^a^	52
Low	8 (7)	41 (37)^ad^	8 (7)	24 (22)	25 (22)	6 (5)^b^	112
High	3 (3)^a^	38 (34)^be^	6 (5)	16 (14)	28 (25)	21 (19)^c^	112
Very High	1 (4)	3 (12)^cde^	1 (4)	4 (15)	6 (23)	11 (42)^abc^	26
Data Collection							
Face to Face	12 (6)	67 (35)	11 (6)	35 (19)	40 (21)	25 (13)	190
Internet	7 (6)	45 (40)	5 (5)	15 (13)	26 (23)	14 (13)	112
Total	19	112	16	50	66	39	302

Figures with the same subscript are significantly different, *p* < .05. Figures in parentheses are percentages calculated for each row

### Material

The survey material consisted of 48 cards describing situations in which the police detained people on suspicion of substance trafficking. Each scenario contained four items of information (a) the age of the person apprehended (a teenager of about 17 years or an older man of about 40 years), (b) the amount of substance found on that person (small or large amount), (c) the type of substance (cannabis, cocaine or heroin) and (d) the charge against him (simple possession of substance, sale of substance to adults, sale of substance to minors or sale at the school gate). Scenarios were obtained by orthogonally crossing these four factors. The design was Age x Quantity x Type of substance x Charge, 2 × 2 × 3 × 4.

An example scenario (translated from Spanish) is as follows: Wilson Ramirez, age 17, was caught by the police in possession of a significant amount of cocaine (enough to make 20 doses). This is the first time Wilson has been arrested. At the time of the arrest, Wilson was selling this amount or part of it to a teenager like himself who appeared to be one of his regular customers. What level of conviction do you think Wilson deserves”? Responses were provided on an 11-point scale with values ranging from No sentence (0) to Extremely severe sentence (10).

### Procedure

Data collection was conducted in 2019 and 2020. The procedure followed Anderson’s guidelines for this type of study [25]. For participants interviewed individually, after an initial meeting on the street, it was agreed to meet at the participant’s home later. Therefore, data collection took place in a quiet room. For participants who participated online, immediately after agreeing to participate, they virtually signed an informed consent form. They then received a link to the SurveyMonkey platform. They were accompanied remotely during the familiarization phase of the survey. Afterwards, they completed all scenarios on their own.

In both conditions, participants needed 25-30 minutes to provide the answers. No participants commented on the number of statements or expressed doubts about the plausibility of the situations presented. A demographic questionnaire was filled out at the end of each session. Some respondents spontaneously voiced their views on the topic; these views were registered.

Ethical approval for the study was granted by Ethics Committee of the Konrad Lorenz University, Bogotá, Colombia. The study conformed to the ethical recommendations of the Colombian Society of Psychology. Total anonymity was preserved, and informed consent was obtained from all participants.

## Results

**Fig. 1:**
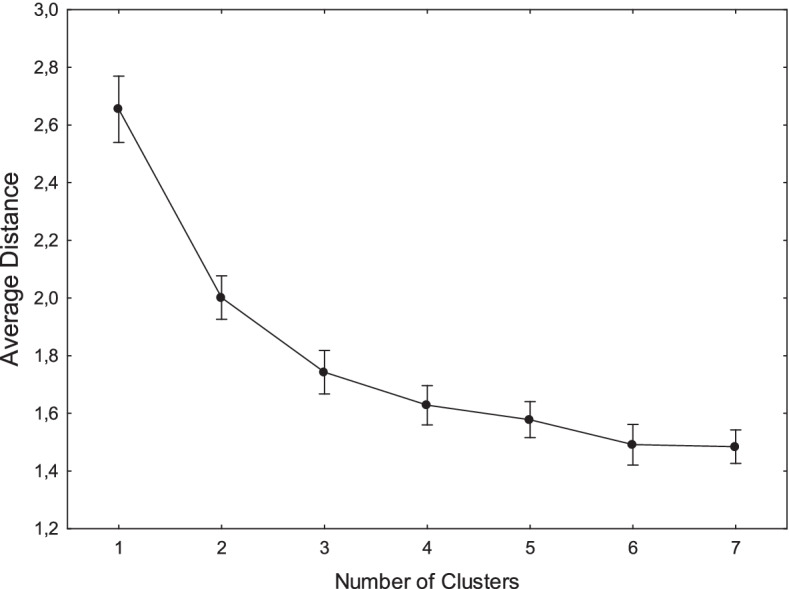
Decrease in the average distance from the centroid as a function of the number of clusters considered

As very widely varying positions were expected, a cluster analysis, using the K-means procedure [26], was performed in order to detect qualitatively different judgment patterns. As four positions were expected, a four-cluster solution was first applied. Subsequently, three-, five-, six-, and seven-cluster solutions were examined. Figure 1 shows the decrease in the average distance from the centroid as a function of the number of clusters considered. The six-cluster solution was the one that seemed optimal.

**Fig. 2:**
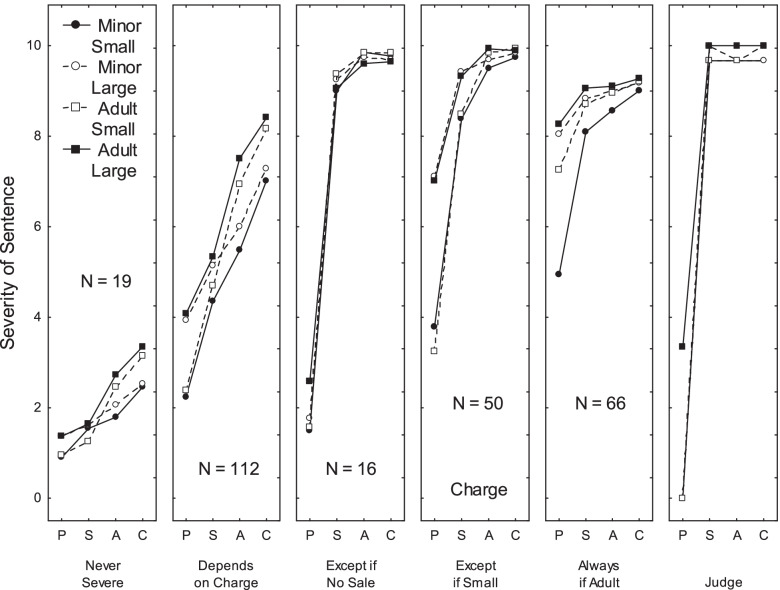
Pattern of ratings observed for five of the six clusters, and for the judge. Each panel corresponds to one cluster. In each panel, the y-axis corresponds to the severity judgments, the x-axis bears the four types of charges, and the four curves correspond to the four combinations of the age factor and of the quality factor

An overall ANOVA was performed on the observed ratings for each profile with a Cluster x Age x Quantity x Substance Type x Charge, 6 × 2 × 2 × 2 × 3 × 4 design. Due to the large number of comparisons, the significance threshold was set at 0.001. The main results are shown in Table 1. Since the Cluster effect and the two-way interaction involving Cluster were significant, six separate analyses were performed at the group level. Figure 2 shows the mean severity scores of five of these six clusters as well as the judge’s ones. The results of the ANOVAs at the group level are shown in Table 2.

**Table 2:** Main Results of the ANOVA

Factor	*df*	*MS*	*F*	*p*	η^2^_p_
Cluster	5	12,286.49	328.03	.001	.85
Age	1	186.98	16.29	.001	.05
Quantity	1	604.77	85.71	.001	.22
Charge	3	5769.65	497.77	.001	.63
Substance	2	104.58	35.79	.001	.11
Age x Charge	3	11.59	3.62	.05	.01
Quantity x Charge	3	217.39	74.87	.001	.20
Cluster x Age	5	74.75	6.51	.001	.10
Cluster x Quantity	5	99.62	14.12	.001	.19
Cluster x Charge	15	694.07	59.88	.001	.50
Cluster x Substance	10	16.47	5.64	.001	.09
Cluster x Age x Charge	15	34.80	10.86	.001	.16
Cluster x Quantity x Charge	15	44.55	15.34	.001	.21

The first cluster (*N* = 19, 6% of the sample) was labelled *Never Severe.* This designation was given because, as can be seen in Fig. 2 (left-hand panel), all mean ratings were low (*M* = 1.95, *SD* = 1.23). When the offender was selling drugs at school gates, the severity of the sentence (*M* = 2.88, *SD* = 1.27) was somewhat higher than when the offender was simply in possession (*M* = 1.15, *SD* = 0.69), η^2^_p_ = .56. As can be seen in Table 1, younger participants or participants with low levels of religiosity expressed this position more frequently than older participants (36+ years) and participants with high levels of religiosity.

The second cluster (*N* = 112, 37%) was labeled *Depends on the charge*. This designation was given because, as can be seen in Fig. 2 (second panel), severity ratings were considerably higher when the offender was selling drugs at the school gates (*M* = 7.72, *SD* = .58) than when he was simply in possession (*M* = 3.17, *SD* = 0.46), η^2^_p_ = .69. In addition, severity ratings were slightly higher (a) when the offender was an adult (*M* = 5.94, *SD* = 0.62) than when the offender was a minor (*M* = 5.18, *SD* = 0.58), η^2^_p_ = .25, (b) when the amount of substance was high (*M* = 5. 96, *SD* = 0.56) than when it was small (*M* = 5.16, *SD* = .55), η^2^_p_ = .45, and (c) when the substance was heroin (*M* = 5.89, *SD* = 0.45) than when it was cannabis (*M* = 5.16, *SD* = 0.47), η^2^_p_ = .32. Older participants or participants with lower socioeconomic status, or participants with children, or participants with a very high level of religiosity expressed this position less frequently than younger participants (49 years or less), participants with higher socioeconomic status, participants without children, and participants with lower levels of religiosity.

The third cluster (*N* = 16, 5%) was labeled *Always severe except in the case of simple possession*. This designation was given because, as can be seen in Fig. 2 (third panel), severity ratings were, irrespective of the context, always considerably lower when the offender was simply in possession (*M* = 1.85, *SD* = 1.04) than when the offender was selling drugs (*M* = 9.56, *SD* = 0.48), η^2^_p_ = .96. Older participants expressed this position more frequently than participants aged 36-49. The pattern of ratings given by the members of this cluster was very similar to that observed in the judge (right panel).

The fourth cluster (*N* = 50, 17%) was labeled *Always severe except in the case of simple possession of small amounts*. As can be seen in Fig. 2 (fourth panel), severity ratings were considerably lower when the offender was simply in possession of small amounts (*M* = 3.51, *SD* = 0.77) than in all other cases (*M* = 9.15, *SD* = 0.28), η^2^_p_ = .58. In addition, severity ratings were slightly higher when the substance was heroin (*M* = 8.65, *SD* = 0.36) than when it was cannabis (*M* = 8.14, *SD* = 0.36), η^2^_p_ = .28. Younger participants expressed this position less frequently than participants aged 29-35.

The fifth cluster (*N* = 66, 22%) was labeled *Always severe for adult dealers*. As can be seen in Fig. 2 (fifth panel), severity ratings were somewhat lower when the offender was a minor who was simply in possession of small amounts (*M* = 4.93, *SD* = 0.37) than in all other cases (*M* = 8.70, *SD* = 0.29), η^2^_p_ = .20. In addition, severity ratings were slightly higher when the substance was heroin (*M* = 8.58, *SD* = 0.44) than when it was cannabis (*M* = 8.31, *SD* = 0.52), η^2^_p_ = .18. Participants aged 36-49 and participants with high socio-economic levels expressed this position more frequently than participants aged 18-28 and participants with low socio-economic level.

Finally, the sixth cluster (*N* = 39, 13%, not shown) was labelled *Always severe.* This designation was given because all mean ratings were very high (*M* = 9.84, *SD* = 0.24). When the offender was selling drugs at school gates, the severity of the sentence (*M* = 9.88, *SD* = 1.12) was slightly higher than when the offender was simply in possession (*M* = 9.71, *SD* = 0.25), η^2^_p_ = .16. As can be seen in Table 1, older participants, female participants, participants with children, participants with very low socio-economic status and participants with a very high level of religiosity expressed this position more frequently than younger participants (49 or less), male participants, participants without children, participants with high socio-economic status, and participants with lower levels of religiosity.

**Fig. 3:**
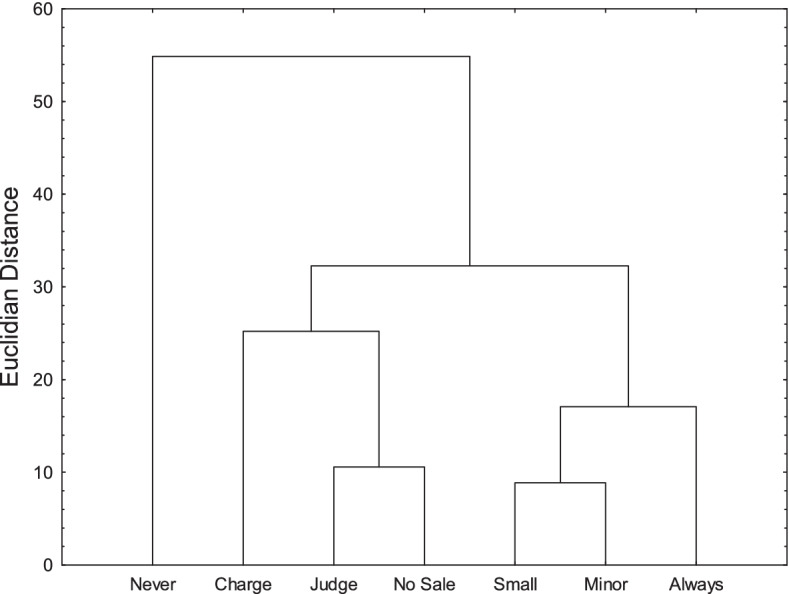
Euclidian distances between the seven observed positions

Figure 3 shows the Euclidian distances between the seven profiles of mean ratings. The main opposition was between the *Never severe* position and all the other positions. There was also a minor opposition between the *Charge* and *No sale* positions on the one hand, and the three *Always severe* positions on the other hand. Unsurprisingly the judge’s personal position was close to the *No sale* position.

## Discussion

As expected, two radical positions were found. For 6 % of the participants, mostly the youngest and least religious, even the sale of illicit substances, by an adult, at the school gates should not result in a very severe sentence. This finding is reminiscent of the one reported by López López and collaborators [21] that 8 % of respondents to a survey on substance control policies expressed the idea that the only acceptable policy is a free market policy. There is, of course, a substantial difference between a free-market policy and a policy of decriminalization of drugs. The common idea, however, is that the possession, use and trade of drugs should not be prosecuted, except perhaps in certain extreme cases.

In contrast, 13% of the participants, mostly older and very religious ones, did not take into account the circumstances in which the protagonist was arrested. From their point of view, even a teenager caught with a personal dose of cannabis should be extremely severely sentenced. This result can be compared with the result reported in the same survey, according to which, for 10% of the participants, only a complete prohibition policy combined with information campaigns about the dangerousness of drugs is acceptable.

Among most participants, however, the positions expressed are seemingly more nuanced. Seventeen percent of the participants expressed a position that is reminiscent of the position defended in 2012 by the Constitutional Court, according to which the mere possession of a personal dose of drugs is not punishable. Twenty-two percent of the participants expressed a position similar to this one, although harsher. In their view, an exception to the maximum severity can only be tolerated in the case of a youth. These results are consistent with those reported by Gritsenko and collaborators [15]. On the other hand, 5% (including one judge) expressed a similar but more flexible position. In their opinion, in all cases where no attempt to sell is proven – cases of simple possession, there is no reason to punish in an extremely severe manner.

Finally, 37% of the participants, mostly younger males without children, and less religious people, expressed a position that punishment should be strictly proportional to the charge against the offender. According to them, an extremely severe punishment is necessary in cases considered serious (e.g., selling at the school gate), mainly if it involves the sale of heroin by an adult, whereas it is not appropriate in cases considered less serious (e.g., simple possession), mainly if it involves the possession of cannabis by a teenager. This position is similar to the one advocated by the International Drug Policy Consortium according to which “proportionate sentencing frameworks should distinguish between the type of drugs and the scale of the illicit activity, as well as the role and motivation of the offender” ([27], p. 1).

The results of the present study are broadly consistent with those of Kirby and Jacobson [14]. The three situational factors – type of substance, amount of substance, and type of charge against the offender – do, taken together, have an effect on the degree of sentence severity deemed appropriate. What the present study shows, moreover, is that (a) these effects are manifested in only some of the participants and not in all of them, and (b) the effect of the factors corresponding to the behavior and age of the protagonist is significantly greater than the effect of the factors corresponding to the substance itself.

The results are also consistent with those of Jorgensen [16]. Of all the demographic characteristics, religiosity has the strongest impact on the positions expressed: 42% of the very religious participants expressed the most drastic position and only 12% the position corresponding to the proportionality rule, whereas, among the not very religious participants, the two percentages are 2 and 58%, respectively.

### Limitations

The main limitation is that the sample was a convenience sample of non-professionals living in one area of Colombia who agreed to respond to a lengthy survey. This study was not epidemiological in nature. As noted above, its purpose was to map, in an exploratory way, people’s opinions about the penalties that should be imposed on people who have been arrested by police for possession of illegal substances, not to determine the exact percentages of people who hold each of these opinions. No major differences were found whether the data were collected via the Internet or face-to-face. Future studies should, using a shortened version of our material, analyze the views of fully representative samples of Colombian adults and compare them to the views expressed by people in other parts of Colombia (e.g., rural areas) and by people in other countries, especially countries with different drug control policies.

## Conclusion

The positions expressed by the participants correspond to three distinct, classical philosophies (a) a libertarian, free-market philosophy – punishment should never be extremely severe because the trade in psychotropic substances is a trade like any other (6%), (b) a moralistic, conservative philosophy – punishment should always be extremely severe except perhaps in certain cases (52%), and (c) a progressive, human rights-inspired philosophy – punishment should always be proportional to the seriousness of the facts (42%).

The fact that the majority of participants expressed a moralistic stance may be related to the realization that successive Colombian governments have never succeeded in establishing a control policy that is not moralistic, i.e., a policy according to which the use of psychotropic substances is considered profoundly immoral, so offenders must be punished in the most dissuasive way possible (regardless of the actual personal and public health consequences of such behavior). It is also related to (a) the judicial polarization that Colombia has experienced, especially in the last 20 years, which has not allowed for the consolidation of strategies against drug possession, and (b) the speeches of different political leaders who have often expressed personal ideologies disconnected from social reality and scientific evidence.

Furthermore, participants expressing non-moralist views are divided. While the majority express views similar to those of international organizations such as the International Drug Policy Consortium, a minority express views that go much further. This fact probably weakens the local relevance of their arguments. There is therefore a concern that any change in legislation in either direction is likely to generate discontent among large segments of society. It is therefore desirable that legislators rely heavily on progressive international legislation [28] to support domestic policies that are not strictly moralistic and conservative.

## Data Availability

All data collected is available and can be accessed by contacting the corresponding author.

## Abbreviations

SD: Standard deviation
ANOVA: Analysis of variance
M: Mean
